# Sodium-Related Adaptations to Drought: New Insights From the Xerophyte Plant *Zygophyllum xanthoxylum*

**DOI:** 10.3389/fpls.2018.01678

**Published:** 2018-11-20

**Authors:** Jie-Jun Xi, Hong-Yu Chen, Wan-Peng Bai, Rong-Chen Yang, Pei-Zhi Yang, Ru-Jin Chen, Tian-Ming Hu, Suo-Min Wang

**Affiliations:** ^1^College of Grassland Agriculture, Northwest A&F University, Xianyang, China; ^2^Noble Research Institute, Ardmore, OK, United States; ^3^State Key Laboratory of Grassland Agro-Ecosystems, College of Pastoral Agriculture Science and Technology, Lanzhou University, Lanzhou, China

**Keywords:** drought, leaf succulence, sodium, xerophyte, *Zygophyllum xanthoxylum*

## Abstract

Understanding the unusual physiological mechanisms that enable drought tolerance in xerophytes will be of considerable benefit because of the potential to identify novel and key genetic elements for future crop improvements. These plants are interesting because they are well-adapted for life in arid zones; *Zygophyllum xanthoxylum*, for example, is a typical xerophytic shrub that inhabits central Asian deserts, accumulating substantial levels of sodium (Na^+^) in its succulent leaves while growing in soils that contain very low levels of this ion. The physiological importance of this unusual trait to drought adaptations remains poorly understood, however. Thus, 2-week-old *Z. xanthoxylum* plants were treated with 50 mM NaCl (Na) for 7 days in this study in order to investigate their drought tolerance, leaf osmotic potential (Ψ_s_) related parameters, anatomical characteristics, and transpiration traits. The results demonstrated that NaCl treatment significantly enhanced both the survivability and durability of *Z. xanthoxylum* plants under extreme drought conditions. The bulk of the Na^+^ ions encapsulated in plants was overwhelmingly allocated to leaves rather than roots or stems under drought conditions; thus, compared to the control, significantly more Na^+^ compared to other solutes such as K^+^, Ca^2+^, Cl^-^, sugars, and proline accumulated in the leaves of NaCl-treated plants and led to a marked decrease (31%) in leaf Ψ_s_. In addition, the accumulation of Na^+^ ions also resulted in mesophyll cell enlargement and leaf succulence, enabling the additional storage of water; Na^+^ ions also reduced the rate of water loss by decreasing stomatal density and down-regulating stomatal aperture size. The results of this study demonstrate that *Z. xanthoxylum* has evolved a notable ability to utilize Na^+^ ions to lower Ψ_s_, swell its leaves, and decrease stomatal aperture sizes, in order to enable the additional uptake and storage of water and mitigate losses. These distinctive drought adaption characteristics mean that the xerophytic plant *Z. xanthoxylum* presents a fascinating case study for the potential identification of important and novel genetic elements that could improve crops. This report provides insights on the eco-physiological role of sodium accumulation in xerophytes adapted to extremely arid habitats.

## Introduction

Drought is the most devastating and commonly occurring global environmental stress that severely impairs food productivity in agriculture and grassland systems (Boyer, 1982; Glantz, 1994; Lambers et al., 2008; Zhao and Running, 2010; Nuccio et al., 2015). This stress is likely to cause more and more issues for food security because predictions suggest that droughts will increase in both severity and frequency given current scenarios for ongoing global climate warming (Li et al., 2009; FAO, 2010; Mishra and Singh, 2010; IPCC, 2014). Thus, understanding the resistance mechanisms used by plants in response to drought is extremely important in light of global and regional changes, not just to forecast the population dynamics of natural ecosystems, but also to enhance agricultural management practices (De Micco and Aronne, 2012).

Species adapted to arid environments tend to survive and grow better during droughts than mesic-adapted counterparts when cultivated together in garden experiments or in natural ecotones (Splunder et al., 1996; Allen and Breshears, 1998; Volaire et al., 1998; McDowell et al., 2008). This attribute is indicative of an underlying difference in the genetic inheritance of drought-resistant traits, as well as the fact that plants with the ability to tolerate conditions where water is scare probably evolved novel mechanisms and key genetic resources which are absent in mesophytes. In light of this background, there has been increasing research interest in revealing the physiological behaviors and mechanisms that underpin the drought adaptations and strategies seen in xerophytes (Yoshimura et al., 2008; Esen et al., 2012; Shi et al., 2013; Yuan et al., 2015), plants that live in arid regions such as the Gobi Desert. Developing a deeper understanding of how these plants manage to resist drought at the physiological level will not only be very beneficial in itself but will also capitalize on the increasing availability of bioinformatics data (Ma et al., 2016; Wu and Su, 2016) and will help to identify the genetic components that are key to crop improvements.

The xerophyte *Zygophyllum xanthoxylum*, a member of the Zygophyllaceae, is a perennial and deciduous succulent shrub that is mainly distributed in the extremely arid regions of northwestern China (Chinese Vegetation Editorial Board, 1980; Ma, 1989; Sheahan and Chase, 2000). The annual precipitation in these areas ranges between 50 and 200 mm, evaporation is more than 2,000 mm per year (Liu and Qiu, 1982), and summer ground temperatures can reach as high as 70°C (Liu et al., 1987); under these conditions, any occasional light rainfall rapidly evaporates. Previous research has shown that *Z. xanthoxylum* accumulates large amounts of Na^+^ ions from the soils where it grows containing extremely low levels of salt (Wang et al., 2004). Therefore, in terms of drought resistance, the function of accumulated Na^+^ in *Z. xanthoxylum* has mostly been attributed to the fact that an increased amount of these ions will contribute significantly to reducing the osmotic potential (Ψ_s_) of leaves (Ma et al., 2012; Yue et al., 2012), thus enhancing the uptake of water during droughts. At the same time, however, the root system of *Z. xanthoxylum* tends to be shallow, distributed in sandy loams within the top 100 cm of the soil profile, mainly at depths between 20 and 40 cm (Chen et al., 2001; Zhou et al., 2006; Kuang et al., 2016); the water content at soil depths between 10 and 20 cm is less than 1%, whereas it ranges between 2 and 3% at depths between 20 and 100 cm (Feng and Cheng, 1999; Sheng et al., 2004). As the matric potential of soils with such an extremely low water content is significantly less than -2.0 MPa (Richards and Weaver, 1944; Wadleigh et al., 1946; Serraj and Sinclair, 2002) and the matric potential decreases approximately vertically in such dry soils if soil water is continuing to be consumed (Serraj and Sinclair, 2002), water absorption by *Z. xanthoxylum* would normally be expected to be impossible via lowering Ψ_s_ by Na^+^ accumulation in its extreme dry habitat. The ability to enhance water uptake via osmotic adjustments due to Na^+^ accumulation may make a limited, though perhaps critical, contribution that enables *Z. xanthoxylum* to live in such extreme habitats. Eco-physiologically, however, why *Z. xanthoxylum* accumulate large quantities of Na^+^ from their natively low Na^+^ content soils or how the accumulated Na^+^ benefits their drought adaptations remains unclear.

This study aims to elucidate the precise role of accumulated Na^+^ in the drought adaptations of *Z. xanthoxylum*. Thus, the effects of these ions on mortality under extreme drought, and on leaf succulence, rate of water loss by transpiration, and stomatal aperture size were all investigated and discussed to elucidate the possible eco-physiological role of the accumulated sodium in *Z. xanthoxylum*.

## Materials and Methods

### Plant Growth Conditions and NaCl Treatments

Seeds of *Z. xanthoxylum* were collected from wild plants within the Alxa League (39°05′N, 105°34′E at an elevation of 1,360 m) in the Inner-Mongolia Autonomous Region, China. Healthy seeds were washed several times under running water using magnetic stirrers to remove any substances that might inhibit their germination, soaked in distilled water for 1 day at 4°C, and germinated for 2 days in the dark at 25°C. Vigorous germinating seeds with uniform radicle length were selected and transplanted into plastic trays (15 cm × 24 cm × 11 cm; 12 seedlings per container) filled with 2-mm diameter white quartz grits. The quartz grits were washed with standard procedure to remove Na^+^ as much as possible: firstly, soaked in 2N sulfuric acid and 1N hydrochloric acid for 2 days, then washed with running water four times, and lastly with distilled water six times. It should be noted that there was still trace amounts of Na^+^ left in washed quartz grits (0.8 ± 0.1 micromoles⋅g^-1^ dry wt.). And this size of grit is too coarse to maintain or absorb water. The plants were irrigated with modified half-strength Hoagland nutrient solution containing 2 mM KNO_3_, 0.5 mM NH_4_H_2_PO_4_, 0.25 mM MgSO_4_⋅H_2_O, 0.1 mM Ca (NO_3_)_2_⋅4H_2_O, 50 μM Fe-citrate, 92 μM H_3_BO_3_, 18 μM MnCl_2_⋅4H_2_O, 1.6 μM ZnSO_4_⋅7H_2_O, 0.6 μM CuSO_4_⋅5H_2_O, and 0.7 μM (NH_4_)_6_Mo_7_O_24_⋅4H_2_O. Prior to planting, the weight of each grit-filled container was recorded using an electronic scale to control and record the proper amount of solution added to each tray daily. In order to prevent drowning damage to roots, the amount of culture solution was limited to depths of between 1 and 2 cm, approximately one-seventh the height of each cultivation tray. Seedlings were grown in a culture room at 32°C/26°C (day/night) under a light/dark cycle of 16 h/8 h and at a light intensity of 400 μm mol m^-2^ s^-1^. Relative humidity was maintained at approximately 65% in growth room.

The culture boxes containing 2-week-old *Z. xanthoxylum* seedlings were randomly separated into two groups and treated for 1 week with modified half-strength Hoagland nutrient solution supplemented with, or without, 50 mM NaCl. The NaCl concentration used in this study was based on previous work where 50 mM NaCl was shown to be optimum among a series of concentration gradients to promote growth and alleviate the deleterious impacts of drought stress (Ma et al., 2012; Yue et al., 2012). Distilled water was supplemented every day to maintain a relatively constant culture solution concentration.

### Survival Rate and Evapotranspiration Assays

In order to determine whether or not NaCl treatment had a positive effect on drought tolerance in *Z. xanthoxylum*, plant watering was curtailed after each treatment. The culture solutions quickly became dried out within 2 days through evapotranspiration, plant deaths occurred following unrecoverable wilt, and the survival rate was calculated as (live plants/total plants) × 100%. In addition, the amount of evapotranspiration was recorded in each case on the 7th day of treatment, calculated as the total weight of each tray containing plants following water supplementation at the end of the sixth treatment day minus the total weight of the tray containing plants at the end of the seventh treatment day.

### Leaf Cell Damage and Antioxidant Systems Assays

In order to further verify the effects of NaCl, a mimic water deficit experiment was performed using -0.5 MPa mannitol (Michel et al., 1983) prepared with half-strength Hoagland nutrient solution. This combination was applied over a 24-h period to plants that had been treated with NaCl for 7 days, and a number of biochemical indicators were assayed using leaves from the third and fourth nodes counted from the bottom of plants (the same below). Thus, the superoxide radical was determined using hydroxylamine oxidation (Elstner and Heupel, 1976), and lipid peroxidation was assessed by monitoring malondialdehyde (MDA) production in leaves, quantified as thiobarbituric acid reactive substances (Dhindsa et al., 1981). Leaf electrolyte leakage was determined by means of relative membrane permeability as measured using a conductivity meter (Murray et al., 1989), while superoxide dismutase (SOD) was assayed using the nitroblue tetrazolium method (Dhindsa et al., 1981), and catalase (CAT) and peroxidase (POD) activities were measured following the method outlined by Cakmak et al. (1993). The ascorbate peroxidase (APX) activity was determined by Nakano and Asada (1981). Glutathione reductase (GR) activity was determined as described by Rao et al. (1996). The Reduced glutathione (GSH) and oxidized glutathione (GSSG) were determined according to Nagalakshmi and Prasad (2001). Briefly, 0.2 g fresh leaves were homogenized by grinder (BioSpec Mini-Beadbeater-96, United States) in liquid N_2_. For MDA, 5 ml 0.1% (w/v) trichloroacetic acid was used to extract the sample and then centrifuged; the resulted supernatant was added 5 ml of 0.5% thiobarbituric acid solution and heated for 10 min at 100°C. After cooling, the precipitate was removed by centrifugation. The absorbance of the sample was measured at 450, 535, and 600 nm. For SOD, CAT, POD, and APX, 8 ml 50 mM sodium phosphate buffer (pH 7.8) was used to extract the sample, centrifuged to get the crude enzyme solution, and then measured accordingly. For GR, the sample extracted by 1.5 ml 100 mM potassium phosphate buffer (pH 7.8), 0.1% (v/v) Triton X-100 and 2 mM MgCl_2_ was centrifuged 10 min at 12,000 *g*. The supernatant was crude extract. For GSH and GSSG, 5 ml of 5% (w/v) sulphosalicylic acid was used to extract and then centrifuged.

### Inorganic and Organic Leaf Solutes and Their Contributions to Ψ_s_

Contents of Na^+^, K^+^, and Ca^2+^ were measured following the method outlined by Wang et al. (2007). Briefly, these ions were extracted from dried plant samples soaked in 100 mM acetic acid at 99°C for 4 h; ion analysis was then performed using a polarization Zeeman atomic absorption spectrophotometer (Hitachi Z-2000, Hitachi High-Technologies Co., Japan). As for chloride (Cl^-^), 0.2 g fresh leaves oven-dried were grinded (BioSpec Mini-Beadbeater-96, United States) into powder, extracted by 1.2 ml ddH_2_O twice within boiled water bath for 2 h, and determined by Mohr titration (Korkmaz, 2001).

Total soluble sugar content was measured using the anthrone method (Dubois et al., 1956; Chow and Landhäusser, 2004). Briefly, 0.2 g samples of fresh leaves were dried, ground in liquid nitrogen, and double-extracted using 4 ml of 80% ethanol by boiling in capped polypropylene tubes at 95°C for 30 min. Extraction supernatants were then centrifuged at 12,000 rpm for 10 min and combined for sugar analysis; each 0.5 ml extraction was diluted in 1.5 ml of distilled water, and 0.5 ml of 2% (w/v) anthrone ethyl acetate reagent was rapidly added, followed by 2.5 ml of concentrated sulfuric acid (H_2_SO_4_). Absorbance was then measured at 630 nm (Hitachi ultraviolet spectrophotometer U-3900, Hitachi High-Technologies Co., Japan) following 10 min of color development in the dark.

The content of free proline was determined using the sulphosalicylic acid method (Bates et al., 1973). Briefly, 0.2 g samples of fresh leaves were dried and homogenized (Mini-Beadbeater-96, Bio Spec Products Inc. United States) in 5 ml of 3% (w/v) sulphosalicylic acid and centrifuged; ninhydrin and glacial acetic acid were then added to the supernatants, and the mixture was heated at 95°C for 60 min in a water bath. Reactions were then stopped using an ice bath, the mixture was extracted using toluene, and the absorbance was read at 520 nm.

The variable Ψ_s_ was measured using a freezing-point osmometer (FM-8P, Shanghai Yida Instrument Co., Japan). Briefly, fresh leaves were frozen and thawed several times in liquid N and then squeezed using a syringe. Leaf sap was then collected, centrifuged, and then used for Ψ_s_ determination at 25°C. These readings (mmol kg^-1^) were used to calculate Ψ_s_ in Mpa, as follows:

$$\Psi_{\text{s}}=-\text{moles of solute}\times \text{R}\times \text{K}.$$

In this expression, *R* = 0.008314 and *K* = 298.8. Similarly, Ψ_s_ values for each inorganic and organic solute were calculated using the Van’t Hoff equation, as follows:

$$\text{Calculated }\Psi_{\text{s}}(\text{COP) }=-\text{nRT.}$$

In this expression, *n* denotes the number of solute molecules, while *R* = 0.008314, and *T* = 298.8. The contributions of each solute to leaf Ψ_s_ were then estimated, as follows:

$$C=\text{COP}/\Psi_{\text{s}}\times 100\%\text{ (Guerrier, 1996).}$$

### Fresh and Dry Weights, Water and Absolute Water Contents, Leaf Volume, and Water Loss Rate Assays

At the end of treatments, whole plants were washed with distilled water and tissue samples were rapidly taken and immediately weighed to determine fresh weight (FW). Additional tissues were oven-dried at 80°C for 72 h and dry weight (DW) was determined. Thus, tissue and absolute water contents were calculated as follows:

$$\begin{array}{l} \text{Water content}=(\text{FW}-\text{DW})/\text{FW}\times 100\%,\text{ and};\\ \text{Absolute water content}=\text{FW}-\text{DW}. \end{array}$$

The measurement method used to calculate leaf volume was as follows:

$$V_{\text{leaf}}=(M_{\text{leaf}}+M_{1}-M_{2})/\rho \text{water}.$$

This equation was taken from the manual of a patented volumenometer (ZL 2015207858757) with a measurement precision of 0.1 mm^3^; in this expression, *V*_leaf_ refers to leaf volume, while *M*_leaf_ is leaf FW, *M*_1_ is the weight of the volumenometer fully filled with pure water, *M*_2_ denotes the weight of this instrument fully filled with pure water plus the leaf, and ρ_water_ is the density of pure water at ambient temperature.

In order to assess the water loss rate, leaves from the same position as those sampled above were carefully excised from the plant with a scalpel and the cut petiole section was sealed with white vaseline to prevent water loss. These cut leaves were then placed on filter paper in plastic dishes in a dark chamber at constant temperature and humidity and their weights were recorded every 4 h. Water loss rates were calculated as follows:

$$\text{Water loss rate}=(FW_{\text{Tn}}-FW_{\text{Tn+1}})/(\Delta T*FW_{\text{Tn}}).$$

In this expression, FW_Tn_ is the weight at each time point, while ΔT denotes the time interval between consecutive measurements.

### Abscisic Acid (ABA) Content Assay

The extraction and purification of ABA from leaves was carried out following previously described procedures (Dobrev and Vankova, 2012). Briefly, an approximately 1 g sample of fresh leaves was homogenized and extracted using a methanol/double-distilled water/formic acid (15:4:1, v:v:v) mixture. This extract was then passed through a SPE C18 column (T1616-2008, Tianjin BoJin Technology Co., Japan) and the eluate was collected and evaporated using a N_2_-pressure blowing concentrator at room temperature in the dark to about one-tenth of its original volume. The residue was then dissolved in 2 mL of 80% methanol and filtered through a 0.45-μm filter, prior to the determination of ABA content using liquid chromatography mass spectrometry (Applied Biosystems PE Sciex API 2000, ABSciex, United States).

### Light and Scanning Electron Microscope (SEM) Observations

Leaves from Control and Na treatments were fixed using 2.5% (v/v) glutaraldehyde in phosphate-buffered saline (PBS, pH 7.4) for 12 h at 4°C. Samples were then washed with PBS, dehydrated using a graded ethanol series, and embedded in LR White resin (London Resin Co., United Kingdom). The resin was then polymerized at 55°C for 3 days, and 0.5 and 0.1 μm sections were cut with a diamond knife using a Leica EM UC7 ultramicrotome (Leica Mikrosysteme GmbH, Austria).

Semi-thin 0.5 μm sections were placed onto glass slides for light microscope observations, stained with 1% (w/v) Toluidine Blue O (with 1% [wt/vol] sodium borate) for 5 min, and observed using an OLYMPUS BX51 system (Olympus Corporation, Japan).

Leaves were fixed with 2.5% (v/v) glutaraldehyde in pH 7.4 PBS for 12 h at 4°C before being re-washed with PBS, dehydrated using a graded ethanol series, immersed in isoamyl acetate, and fully dried with a critical point dryer for SEM observations. Treated samples were then mounted on copper stubs and sputter-coated with gold-palladium; specimens were observed and photographed using a field emission (FE) SEM (S-4800, HITACHI, Japan) at 10 kV.

### Statistical Analysis

Each experiment was repeated at least twice and comprised three replicates. All values are reported in this study as means ± standard deviation (SD); analysis of variance (ANOVA) was performed for experiments using the software Excel 2016 applying the Student’s *t*-test for simple one-way comparisons of two samples.

## Results

### Application of 50 mM NaCl Enhanced Survival Rate and Durability Under Extreme Drought Conditions

**FIGURE 1:**
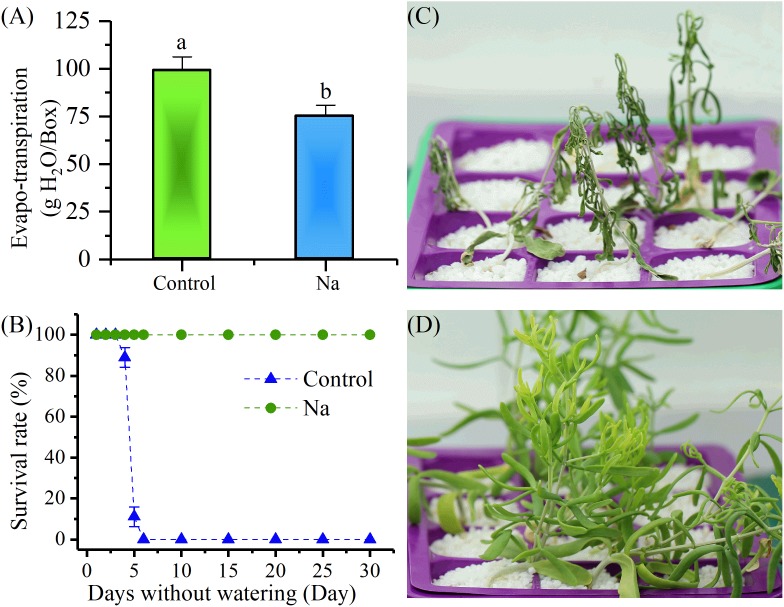
NaCl treatment (50 mM) for 7 days increased the survival rate and durability of 2-week-old *Zygophyllum xanthoxylum* seedlings compared to controls when water was withheld. **(A)** Water lost by evapotranspiration per box on the 7th day of NaCl treatment. **(B)** Survival rate versus days that water was withheld; **(C,D)** show representative photographs of control and NaCl-treated plants taken after 19 days without water, respectively. Bars denote the mean ± SD of between three and four replicates; each replicate in **(A)** involved 12 plants. Bars with different letters are significantly different at the level of 0.05.

Two-week-old *Z. xanthoxylum* seedlings were randomly treated either with NaCl, or without NaCl (control), for 1 week. The results revealed that water loss by evapotranspiration from trays of NaCl-treated plants was significantly less than from control plants on the 7th day after treatment (Figure 1A). Watering was stopped following treatment and time-course analyses of survival rate and durability were performed. These experiments showed that because of rapid evapotranspiration and the fact that large quartz grit particles cannot retain moisture, the solution quickly ran out in the culture trays within 2 days or less. On the 3rd day after watering was stopped, the control plants started to visibly wilt; as shown in Figure 1B, the survival rate of this group also sharply decreased between the 4th and 6th day after watering was stopped because of unrecoverable wilt, while this rate did not change in the NaCl- treated group. It is also noteworthy that the plants in the control group were totally dried out by between 6 and 8 days after watering was stopped, while this took between 35 and 45 days in the treatment group (Figures 1C,D). The results corroborated the fact that NaCl treatment enhanced the survival rate and durability of *Z. xanthoxylum* under absolute drought conditions.

### Treatment With 50 mM NaCl Enhanced Adaptability to Short-Term Osmotic Stress

**FIGURE 2:**
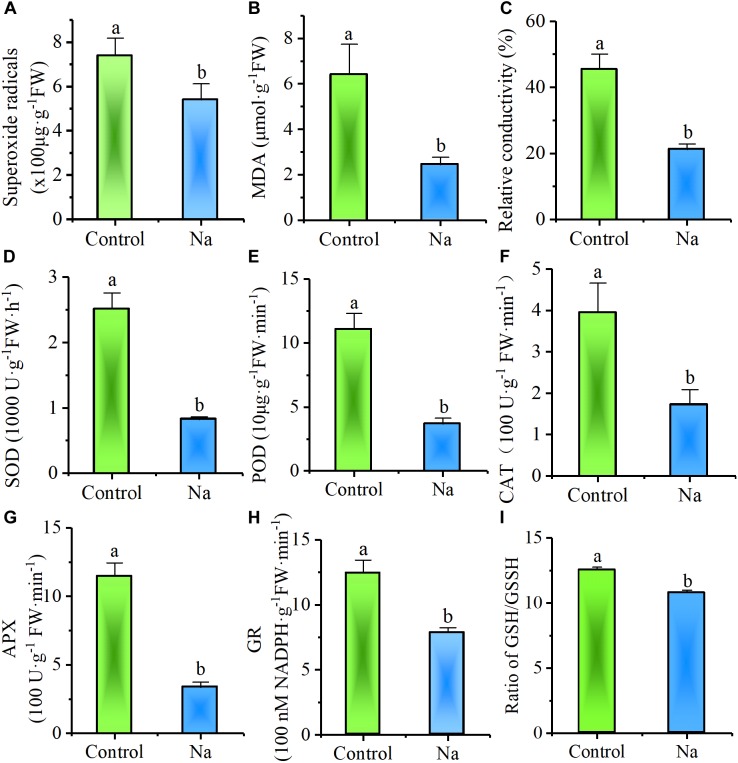
NaCl treatment reduced *Z. xanthoxylum* leaf damage from short-term osmotic stress induced with -0.5 MPa mannitol. Relative biochemical content of **(A)** superoxide radicals, **(B)** MDA, **(C)** electrolyte leakage and antioxidase activity of **(D)** SOD, **(E)** POD, **(F)** CAT, **(G)** APX, **(H)** GR as well as **(I)** Ratio of GSH/GSSH were all analyzed in leaves harvested after 24 h of –0.5 MPa mannitol stress from third and fourth nodes of 2-week-old *Z. xanthoxylum* seedlings treated with 50 mM NaCl or without (control) for 7 days. Bars denote the mean ± SD (*n* = 8), with different letters indicating significantly different at the level of 0.05.

In order to test whether, or not, NaCl treatment enhances osmotic stress resistance, plants that had been subject to 7 days of treatment were exposed for 24 h to a further culture solution containing an additional -0.5 MPa of mannitol. Observations revealed that less of this solution remained in the control trays than in those treated with NaCl, while visible wilt wrinkles appeared on the leaves of control plants but not on treated individuals. Consistent with these observations, biochemical assays demonstrated that the content of the superoxide radical (O_2_^-^⋅), a reactive oxygen species (ROS) produced in plants under stress, was significantly less in NaCl-treated plants than in the control group (Figure 2A). In addition, the content of MDA, an end product of lipid peroxidation that is stimulated by ROS, was also significantly lower in NaCl-treated plants (Figure 2B), while the relative conductivity or electrolyte leakage, proxy for the degree of cell membrane damage due to stress, was also markedly reduced in NaCl-treated plants (Figure 2C). The activity of enzymes involved in removing ROS, such as SOD, POD, CAT, APX, and GR were also significantly reduced in NaCl-treated plants (Figures 2D–H). The Ratio of the non-enzymatic antioxidant reduced/oxidized glutathione (GSH/GSSG) was lower in NaCl-treated plants, too, indicating the treated leaves with more efficiency to use reduced glutathione (Figure 2I). All of these results demonstrated that treatment with NaCl reduced the damage caused by osmotic stress.

### Na^+^ Contributed to, and Significantly Decreased, Leaf Ψ_s_

**FIGURE 3:**
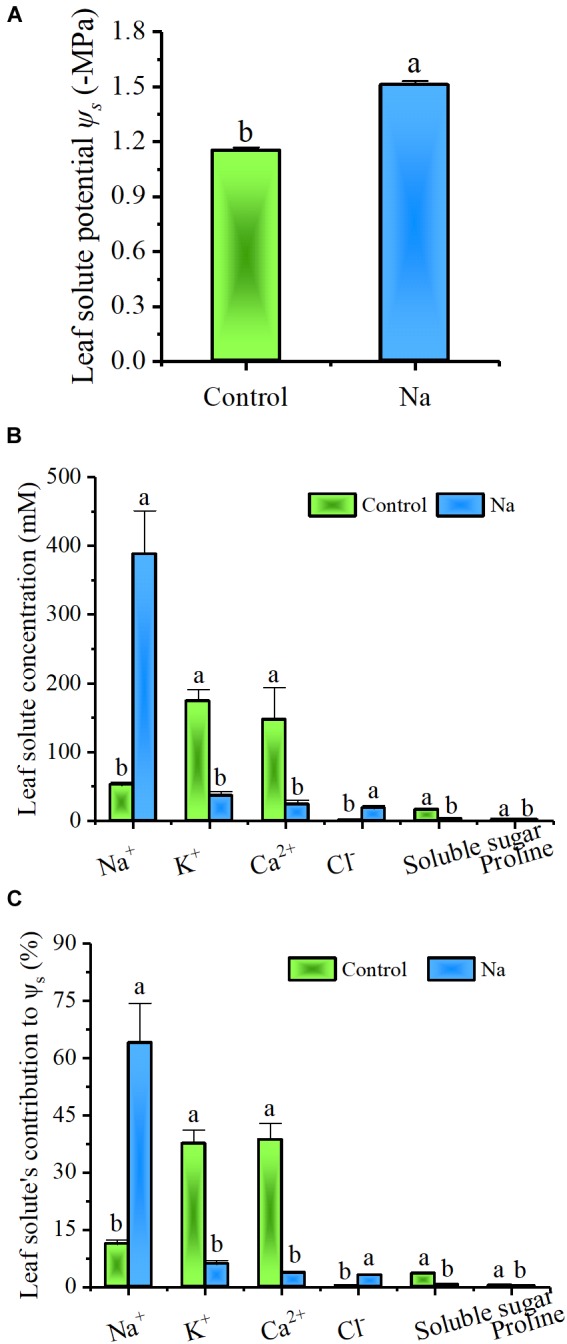
Na^+^ not only significantly decreased Ψs but made a large contribution to Ψs in leaves from the third and fourth nodes in 2-week-old *Z. xanthoxylum* seedlings treated with 50 mM NaCl or without (control) for 7 days. **(A)** (Ψs). **(B)** Solute concentration in leaves. **(C)** Solute contributions to Ψs. Bars denote the mean ± SD (*n* = 8), with different letters indicating significantly different at the level of 0.05.

In order to investigate the function of Na^+^ in drought tolerance, leaf solute potential (Ψ_s_) and the contents of Na^+^, K^+^, Ca^2+^, Cl^-^, soluble sugar, and proline in leaves were measured. The concentration of each solute, as well as its relative contribution to Ψ_s_, were also calculated. Results showed (Figure 3A) that Ψ_s_ was significantly lowered in NaCl-treated plants compared to the control group and the same was true for K^+^, Ca^2+^, soluble sugar, and proline, while opposite pattern was seen in Na^+^ and Cl^-^ content (Figure 3B). Na^+^ was greatly accumulated to more than 50 mM in control group plants (Figure 3B), even though these ions were not added to the culture solution and a great deal of effort was expended to clean the quartz grit used in these experiments. At the same time, the contribution of inorganic solutes, such as Na^+^, K^+^, Ca^2+^, and Cl^-^, to Ψ_s_ was as high as 87.36% in the control plants, while the contribution of Na^+^ was just 11.2% ± 1.1%. In treated plants, the contribution of inorganic solutes was 76.37%, and the Na^+^ contribution was definitely higher, 63.9% ± 10.4% (Figure 3C). These results suggested that inorganic solutes of Na^+^, K^+^, Ca^2+^, and Cl^-^ are the preferred osmolytes of *Z. xanthoxylum* and that the first of them is favored overall. Thus, when available, Na^+^ is rapidly accumulated in leaves, significantly decreasing their solute potential.

### Treatment With 50 mM NaCl Enhanced Leaf Succulence and Water Storage

**FIGURE 4:**
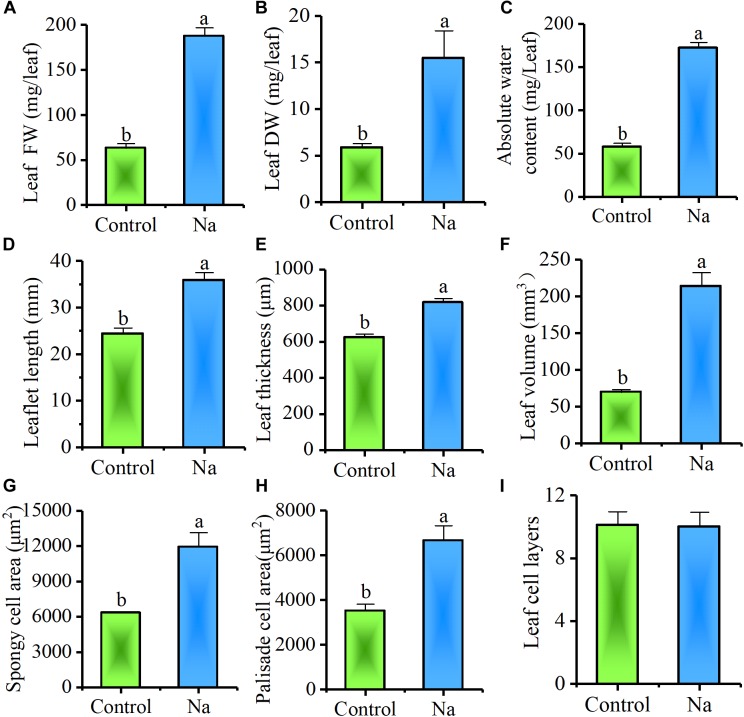
Treatment with NaCl promotes leaf succulence. The samples for this experiment were third- and fourth-node leaves from 2-week-old *Z. xanthoxylum* seedlings treated with 50 mM NaCl or without (control) for 7 days. These results show that NaCl treatment stimulated **(A)** leaf FW, **(B)** DW, and **(C)** absolute water content **(D)**, as well as leaflet length **(E)**, leaf thickness **(F)** and volume **(G)**, and spongy **(H)** and palisade cell area, but not **(I)** leaf cell layers. Bars denote the mean ± SD (*n* = 8), with different letters indicating significantly different at the level of 0.05.

In order to further determine the effects of accumulated Na^+^ on leaf growth, mature leaf characteristics pruned from the third and fourth nodes of plants were examined. The results showed that leaf FW and DW, absolute water content, leaflet length, thickness, and volume all markedly increased in treated plants (Figures 4A–F). Results also suggested that NaCl treatment had a great impact on promoting leaf succulence.

**FIGURE 5:**
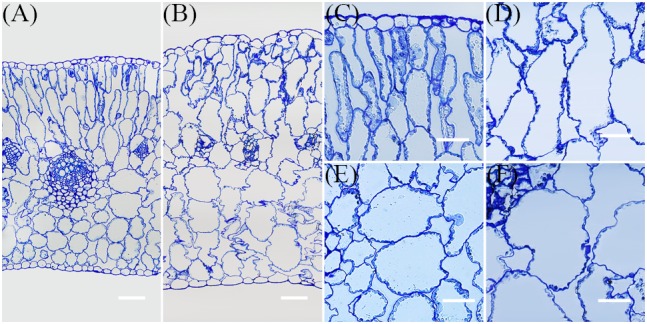
Representative transverse semi-thin sections of leaves. The samples for this experiment were third- and fourth-node leaves from 2-week-old *Z. xanthoxylum* seedlings treated with 50 mM NaCl or without (control) for 7 days. All these sections were stained with toluidine blue. Semi-thin section of leaf from control **(A)** and NaCl treatment **(B)**. Palisade cells from control **(C)** and NaCl treatment **(D)**. Spongy cells from control **(E)** and NaCl treatment **(F)**. The scale bars in **(A,B)** are 100 μm, while those in **(C–F)** are 50 μm.

Further, to examine the effect of NaCl on leaf succulence at the cellular level, semi-thin cross-sections of mature leaves from the same positions on plants were investigated. Compared with the control, both palisade and spongy cells were enlarged dramatically in NaCl-treated plants (Figure 5); because of cell enlargement, morphological differences between palisade and spongy tissues became less obvious in treated plants (Figures 5A,B). Thus, to quantify the effect of Na^+^ on cell enlargement, areas of the three largest palisade and spongy cells in each different section were measured. These results demonstrated that the areas of these cells were significantly increased by 91.3% ± 18.8% and 89.2% ± 19.5% in NaCl-treated plants compared with the control, respectively (Figures 4G,H, 5C–F). Because the number of leaf cell layers did not differ between the control and treatment (Figure 4I), these results suggested that succulence promoted by Na^+^ was mainly the result of cell enlargement rather than cell division.

**FIGURE 6:**
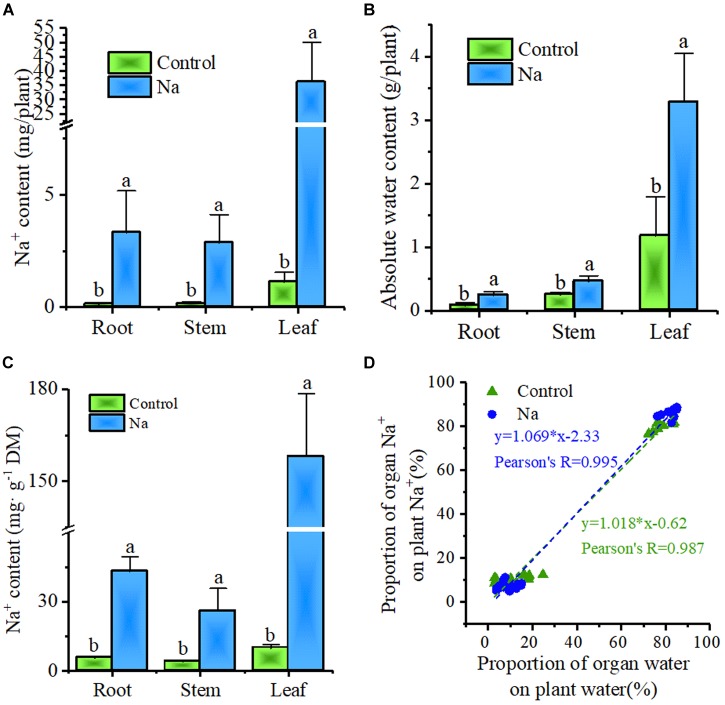
Plant water distribution is positively correlated with plant Na^+^ distribution in root, stem, and leaf organs. The samples for this experiment were third- and fourth-node leaves from 2-week-old *Z. xanthoxylum* seedlings treated with 50 mM NaCl or without (control) for 7 days. **(A)** Na^+^ content in root, stem, and leaf organs. **(B)** Absolute water content in root, stem, and leaf organs. **(C)** Na^+^ concentration in root, stem, and leaf organs. **(D)** Correlation analysis between the proportional distribution of Na^+^ and water in different organs across the whole plant. Bars represent the mean ± SD (*n* = 8), with different letters indicating significantly different at the level of 0.05.

In order to gain a deeper understanding of the significance of Na^+^ in *Z. xanthoxylum*, the distributions of both water and Na^+^ at the organ level in plants were also investigated. These results showed that Na^+^ sequestered in plants was overwhelmingly allocated to leaves, irrespective of treatment or control (Figure 6A), and that the same distribution pattern was seen for water in different organs (Figure 6B). And the Na^+^ concentration was the highest in leaves (Figure 6C). Correlation analysis showed that the Na^+^ distribution varies across organs at the whole plant level, but was positively correlated with the corresponding water distribution (Figure 6D). Thus, absolute plant water content increased by 188.0% ± 33. 5% in NaCl-treated plants compared to the control and that this increment comprised as much as a 99.4% ± 19.0% increase in leaves. These results also showed strongly that Na^+^ absorbed by *Z. xanthoxylum* was mainly allocated to leaves so that cells could be enlarged for water storage.

### Treatment With 50 mM NaCl Decreased Leaf Water Loss and Stomatal Aperture Size

**FIGURE 7:**
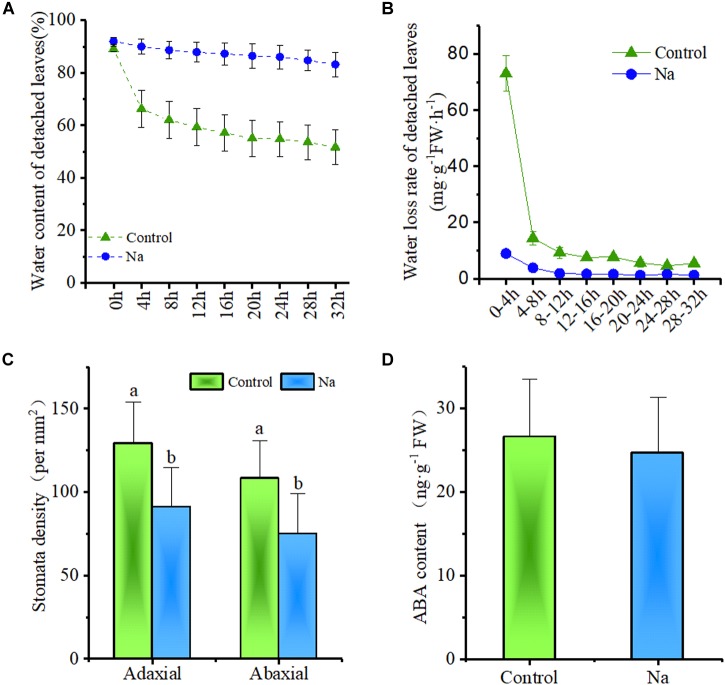
Treatment with NaCl reduced the rate of water loss and stomatal density in detached leaves but did not influence ABA content. The samples for this experiment were third- and fourth-node leaves from 2-week-old *Z. xanthoxylum* seedlings treated with 50 mM NaCl or without (control) for 7 days. **(A)** Water content of detached leaves versus length of time (h) detached (*n* = 8). **(B)** Water loss rate of detached leaves (*n* = 8). **(C)** Stomatal density on adaxial and abaxial leaf surface (*n* = 18). **(D)** Leaf ABA content (*n* = 4). Bars denote the mean ± SD (*n* = 8), with different letters indicating significantly different at the level of 0.05.

Because the results of this study showed that water loss via evapotranspiration at the plant tray level was reduced by NaCl treatment (Figure 1A), it was further hypothesized that this might also act to reduce the plant water loss rate. Thus, to test this, the water loss rates of detached third and fourth node leaves from 2-week-old seedlings treated for 7 days with NaCl and without NaCl (control) were investigated (Figure 7). The results of this comparison showed that when leaves were detached from their parent plant their water content generally declined; however, the rate of water content loss was clearly less in NaCl-treated leaves than in those from control plants (Figure 7A). Results showed that the water loss rate of detached leaves from NaCl-treated plants was only about one eighth of that from their control counterparts after a period of up to 4 h (Figure 7B) and that the rate in leaves from the latter remained significantly higher throughout this period even though overall water content was lower (Figure 7A,B). This suggested that the water loss rate of detached leaves was independent of cell sap concentration; this rate also fell to almost zero in NaCl-treated detached leaves within 12 h, while that in control leaves continued to decline (Figure 7B). This suggested that NaCl treatment promoted water retention and greatly reduced leaf water losses.

**FIGURE 8:**
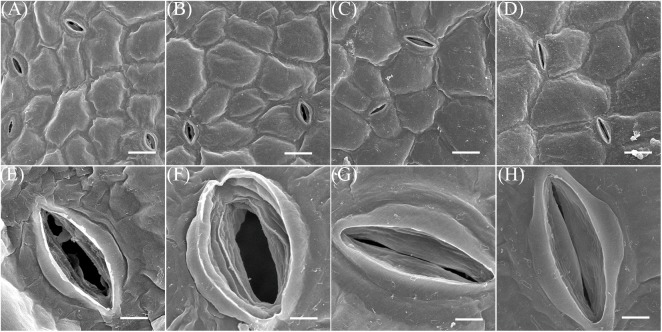
SEM images. The samples for this experiment were third- and fourth-node leaves from 2-week-old *Z. xanthoxylum* seedlings treated with 50 mM NaCl or without (control) for 7 days. **(A,E)** Adaxial stomata in control plants. **(B,F)** Abaxial stomata in control plants. **(C,G)** Adaxial stomata in NaCl-treated plants. **(D,H)** Abaxial stomata in NaCl-treated plants. Scale bars in **(A–C)**, and **(D)** are 20 μm, while those in **(E–H)** are 4 μm. Scanning electron microscope (SEM) analysis of leaves on the 3rd–4th nodes of 2-week *Z. xanthoxylum* seedlings treated without (Control) or with 50 mM NaCl (Na) for 7 days. **(A,E)** Adaxial stomata in control plants. **(B,F)** Abaxial stomata in control plants. **(C,G)** Adaxial stomata in Na treatment plants. **(D,H)** Abaxial stomata in Na treatment plants. Scale bar: **(A–D)** 20 μm; **(E–H)** 4 μm.

To further understand how this treatment influences water loss, leaf stomata from the third and fourth nodes of 2-week-old plants subjected to treatment with, and without, NaCl for 7 days were examined via SEM. These observations showed that although the stomata of control group leaves were mostly open, those on both the adaxial and abaxial surfaces of treatment group plants were closed (Figure 8). Further SEM analysis revealed that 69.5 and 94.4% of adaxial and abaxial epidermal stomata were open in control plants, respectively, while just 5.7 and 6.1% were open in their NaCl-treated counterparts (Supplementary Table S1). Statistical analysis also showed that stomatal density on both the adaxial and abaxial surfaces of NaCl-treated plants was significantly reduced compared to the control (Figure 7C); however, as these leaves were completely developed on the third and fourth nodes of 2-week old-seedlings, this decrease in stomatal density might also be due to the enlargement of epidermal cells in treated plants (Figures 5A,B, 8A–D). Taken together, these results demonstrated that NaCl treatment influenced water loss reduction mainly via stomatal closure and decreases in the density of these structures.

It is likely that levels of the stress signaling molecule ABA, which promotes stomatal closure, increase when plants are subject to water stress. Thus, to detect whether, or not, reductions in stomatal aperture size can be linked to changes in the proportions of this molecule, ABA content was measured. The data showed that ABA content did not vary significantly between the control and NaCl-treated plants (Figure 7D); this suggested that the leaves of NaCl-treated plants may not suffer stress and that stomatal closure was probably independent of ABA.

## Discussion

Droughts occurring in arid regions, or generally because of insufficient rainfall, have always been the main factor limiting crop production across most of the world (Steduto et al., 2012). However, in extremely arid environments, such as the deserts of northwestern China, succulent xerophytes such as *Z. xanthoxylum* manage to grow well. These plants are important because they possess outstanding ability to thrive in arid areas in which model and agronomic plant species cannot survive, and thus they may have evolved a range of novel strategies to cope with drought. Unfortunately, most of the plants that have been the focus of detailed research to date have limited tolerance to drought, including *Arabidopsis* (Clauw et al., 2016), tobacco (Xie et al., 2016), rice (Todaka et al., 2017), wheat (Sukumaran et al., 2016), and maize (Wang et al., 2016). Thus far, relatively little attention has been focused on plants which are already adapted for growth in conditions of severe drought, due largely to the difficulties of working with these slow-growing species. As a result, however, the precise physiological mechanisms exhibited by these drought-tolerant plants remain to be elucidated.

The subject of this paper, the xerophyte *Z. xanthoxylum*, possesses this ability as it could live in the Gobi Desert of northwestern China (Liu et al., 1987). In earlier work, Wang et al. (2004) noted that this plant accumulates abundant Na^+^ from non-saline soils even though its natural habitats are characterized by very low salt content; this suggests that this ion might exert a positive influence on drought tolerance in this species. The results of this study showed that treatment with 50 mM NaCl not only enhanced the survival rate and durability of *Z. xanthoxylum* under extreme drought conditions, but that the presence of this ion also increased the adaptability of this plant to short-term osmotic stress (Figures 1, 2). These results highlighted the fact that Na^+^ is an important component of the desert adaptations seen in *Z. xanthoxylum*.

Increased water uptake in plants can be achieved by decreasing Ψ_s_ via the enhanced accumulation of solutes during droughts or periods of salinity, a process termed osmotic adjustment (Turner and Jones, 1980; Verslues et al., 2006). This adaptation is seen in numerous plant species and is well known to play a role in combating dehydration (Blum, 2017). Indeed, the treatment with 50 mM NaCl applied in this study to *Z. xanthoxylum* significantly decreased leaf Ψ_s_ compared to the control (Figure 3A), a reduction that was overwhelmingly due to increased Na^+^ accumulation (Figures 3B,C). Halophyte plants that live on seacoasts or in saltmarshes, like *Suaeda maritima* (Yeo and Flowers, 1980) and *Atriplex vesicaria* (Black, 1960), also accumulate large quantities of this ion in their leaves to enable enhanced water uptake so as to counteract the external osmotic stresses imposed by the high salt concentrations in their environments (Flowers et al., 1977). However, unlike halophytes that live in habitats where there is plenty of water, this resource is almost entirely absent from the upper desert layer and thus increasing water uptake via Na^+^ accumulation cannot play a great role in the adaptations of *Z. xanthoxylum* in deserts. It is because that water uptake via osmotic adjustments can only function at the onset of drought or during mild-to-moderate events of this kind, in the first place because soil water potentials become far too low to extract additional water during long periods of drought. This is especially the case in soil horizons below depths of 100 cm in arid regions (Nobel, 1977; von Willert et al., 1992). In addition, it is also possible that water potential can drop sharply in severely arid soils if any depletion occurs which further acts to limit the uptake of plants (Serraj and Sinclair, 2002). This conclusion is corroborated by the results of this study because in experiments in which watering was withheld, the Ψ_s_ in control plants fell to lower levels than in NaCl-treated individuals because of the passive decrease associated with wilting. Nevertheless, plants in the control group still died faster and earlier because of no water available (Figure 1). Our results also showed that at the start of the short-term osmotic stress experiment reported in this study, the total calculated Ψ_s_ values of the culture solutions were -0.52 and -0.76 MPa for the control and treatment, respectively; data showed that although control plants should tend to suffer less, they were nevertheless damaged to a greater extent because of a rapid decrease in the Ψ_s_ of the culture solution due to fast water depletion (Figure 2). Thus, in terms of long-term survival, the role of Na^+^ in osmotic adjustment may only contribute to the desert adaptations of *Z. xanthoxylum* to a limited extent because most of the growing season for this plant coincides with extreme drought conditions.

The swelling of tissues and organs for water storage, a process known as succulence, is another important adaptation seen in a diverse range of plant species that cope with semi-arid and arid environments (Ogburn and Edwards, 2010; Males, 2017). This process of tissue and organ swelling involves an increase in cell volume accompanied by the intake of vast amounts of water. Water uptake by cells is generally associated with, or modulated by, cell wall relaxation to reduce turgor pressure (Ψ_p_), modification of cell solute content, and changes in plant hydraulic conductance; the last of these factors, however, only works in cells that are far from their water potential equilibrium (Cosgrove, 1993). This means that water uptake into a cell is mainly controlled by cell wall elasticity and solute content, themselves affected by the Ψ_p_ and Ψ_s_, respectively (Steudle et al., 1977). In a rigid plant cell, Ψ_p_ is primarily dependent on cell solute content; thus, any increase in this variable will result in a concomitant Ψ_p_ increase, leading to a large tensile stress on the cell wall. If a cell wall is in an “extensible” state, it will undergo turgor-driven expansion and water uptake (Cosgrove, 2000). The Ψ_s_ values measured in this study (Figure 3A) showed that total solute content by concentration increased by around 31.3% in leaves of NaCl-treated plants, compared to the control group, a result that was consistent with a previously reported 28.8% increase in cell Ψ_p_ in similarly treated plants (Ma et al., 2012). In contrast, if the leaf cell size of NaCl-treated plants is reduced to the same size as the control group (Figure 5), between two and four times more solute concentration is generated, based on estimates for cell wall increases (Figures 4G,H). This result suggested that cells in the leaves of NaCl-treated plants underwent both turgor-driven expansion and water uptake. The data presented in this study also showed that of the accumulated solutes analyzed, Na^+^ comprised 86.2% of the total in leaves of plants treated in this way (Figures 3B,C); this means that this ion made a major contribution to the increase in Ψ_p_ and cell size. The NaCl treatment therefore notably enhanced water storage by promoting leaf succulence in *Z. xanthoxylum* through cell enlargement (Figures 4, 5). This leads to a number of inevitable conclusions, including that the majority of Na^+^ contained in plants is allocated to leaves, the water distribution at the individual level is tightly positively correlated to the distribution of this ion in different organs, and the prominent rise in absolute water content in NaCl-treated plants was nearly all the result of water increase from leaves (Figure 6). We therefore concluded that the role of absorbed Na^+^ in *Z. xanthoxylum* is mainly to promote leaf succulence by enlarging cells for enhanced water storage. This resource, once stored, helps to mitigate drought stress in tissues and buffer stomatal conductance and photosynthesis over a period of days in the face of soil water deficits (Sinclair, 1983; Martin, 1994). The resultant increase in leaf hydration recorded in this study was also partially responsible for enhanced durability and reduced cell damage (Figures 1, 2) and in addition improved photosynthetic performance under drought stress (Ma et al., 2012; Yue et al., 2012).

Water retention by down-regulating transpiration is another common strategy utilized by plants to sustain the balance of water resource as a drought resistance adaption. This can be achieved via the formation of xeromorphic traits including leaf shedding, a lower leaf number, smaller leaf size, and the presence of xeromorphic leaves with features such as thick cuticles, and stiff or sunken stomata in plants that live in arid and semiarid environments (De Micco and Aronne, 2012). This adaptation can also result from instant adjustments in the stomatal aperture of most plants when subject to water shortages (Sperry, 2000; Liu et al., 2005). This water-saving process could be fulfilled in our study via intensive accumulation of Na^+^ in leaves which consequently resulted in a marked decrease in water loss rate the via down-regulation of stomatal aperture size and decrease in density (Figures 7, 8). This is perhaps one major reason why *Z. xanthoxylum* plants are able to withstand many days under conditions of extreme drought (Figure 1) or are adapted to their desert habitats because of lower water demand and expenditure. As regards stomatal closure, various studies demonstrated that ABA produced in root tips in response to drought in drying soils is a major signaling molecule transported into leaves via the transpiration stream. When this molecule reaches a leaf it leads to an increase in ABA and reduced stomatal conductance (Davies and Zhang, 1991; Davies et al., 1994). The NaCl-treated plants in our study, however, did not suffer from water shortage and so the content of this stress signaling molecule (ABA) was not difference in control and treated plant leaves (Figure 7D); this indicated that stomatal closure in NaCl-treated plants did not result in detectable ABA changes. Additional studies on the stomatal behavior of isolated epidermis have shown that Na^+^ can directly inhibit the opening of these structures in the non-succulent halophyte *Aster tripolium*, and that this process is not mediated by ABA (Perera et al., 1994). Compared to the contrasting and unusual stomatal response of *A. tripolium*, Na^+^ appears to promote opening in non-halophyte and glycophyte species such as *Commelina communis* and *A. amellus* (a relative of *A. tripolium*), finally leading to irreversible opening, disrupting the ability of stomata to close in response to both environmental signals (i.e., darkness and CO_2_) and ABA (Jarvis and Mansfield, 1980; Zeiger, 1983; Perera et al., 1994). The presence of this notable distinction in Na^+^ effects on stomatal behavior between halophytes and glycophytes mainly relies on the fact that the former has evolved a novel mechanism to sense Na^+^ signaling and then to downregulate K^+^ uptake, as the latter is the main ion involving in stomatal opening (Perera et al., 1997; Véry et al., 1998). The mechanism by which Na^+^ downregulates stomatal aperture size could also decrease transpiration and limit the excessive upward passage of this ion in the xylem to the shoots, enabling *A. tripolium* to avoid toxicity in its saline habitat (Kerstiens et al., 2002). It is likely that a similar mechanism has also evolved in the xerophyte *Z. xanthoxylum*. However, unlike halophytes that inhabit saline environments, the species studied in this research lives in soils that contain very low Na^+^ concentrations and also has a marked ability to absorb and distribute large concentrations of this element in its leaves. This means that the active accumulation of larger quantities of Na^+^ in leaves acts to downregulate water loss by transpiration, an additional arid habitat adaptation. This result implies that the xerophyte *Z. xanthoxylum* possesses some unique molecular systems that are distinct from both halophytes and glycophytes.

An ever-increasing body of research demonstrates that, in addition to reduced transpiration, decreased stomatal opening could also result in a lower influx of stomatal CO_2_ and increased temperatures inside leaves, limiting photosynthesis (Sharkey, 2005; Allakhverdiev et al., 2008; Farooq et al., 2012). The results of this study show, however, that both the FW and DW of NaCl-treated plants, and especially of their leaves, increased markedly in comparison to control plants (data not shown). Similar results also have been reported in other studies (Ma et al., 2012; Yue et al., 2012), and provide insights into the presence of possible additional mechanisms in *Z. xanthoxylum* that might act to neutralize the adverse effects on photosynthesis of reduced stomatal opening. One critical mechanism to separate the excess Na^+^ form cytosol in leaf cell should exist, because lots of important metabolic enzymes like malate dehydrogenase, aspartate transaminase, glucose-6-phosphate dehydrogenase, isocitrate dehydrogenase, phosphoribose isomerase, lactate dehydrogenase, nitrate reductase, and 6-phosphogluconate dehydrogenase are all sensitive to high Na^+^ concentrations (Flowers et al., 1977). And most likely, the much Na^+^ accumulated in leave of *Z. xanthoxylum* is localized in vacuole (Flowers et al., 1977; Wu et al., 2011).

**FIGURE 9:**
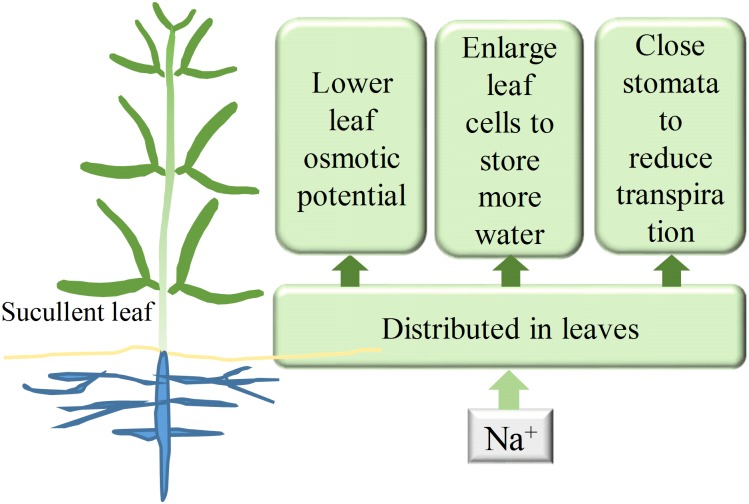
The probable roles of Na^+^ in *Z. xanthoxylum* drought tolerance. A great deal of Na^+^ resulted in a decrease in leaf Ψ_s_ and facilitated more water uptake. Accumulated Na^+^ in leaves enhances water storage by enlarging leaf cells and reduces transpiration by closing stomata. The three possible physiological roles of Na^+^ shown in this figure are likely to enable the xerophyte *Z. xanthoxylum* to thrive in extremely arid habitats.

Drought tolerance in plants depends on the maximization of water uptake and storage and the minimization of water loss (De Micco and Aronne, 2012). The results presented in this study showed that the xerophyte *Z. xanthoxylum* achieves these three aims simultaneously by accumulating Na^+^ in its leaves by decreasing cell Ψ_s_, enhancing leaf succulence, and down-regulating transpiration (Figure 9). Similar strategies may also be employed by leaf-succulent shrubs including *Nolana mollis*, *Heliotropium pycnophyllum*, and *Tetragonia maritima* that are native to the Atacama Desert, as they also accumulate extremely high levels of Na^+^ in their leaves (Rundel et al., 1980). These strategies are important because this desert in northern Chile in one of the two most arid regions globally, with a mean precipitation of less than 25 mm year^-1^. Acquiring and storing water using these approaches is optimal and adaptive because brief precipitation is exhausted quickly, usually within 2 or 3 days, by the scorching summer desert sun (Feng and Cheng, 1999).

## Conclusion

The results of this study demonstrated that Na^+^ can significantly increase the survivability and durability of the xerophyte *Z. xanthoxylum* under drought conditions. These drought adaptations are physiologically most likely the result of high concentrations of Na^+^ distributed in leaves that act to lower Ψ_s_, swell leaf organs, and decrease stomatal aperture size, enabling enhanced water uptake and storage and reducing losses. This intriguing ability to utilize Na^+^ to balance the water budget as an adaptation to life in arid areas suggests that the xerophyte *Z. xanthoxylum* will be a key species for further study, especially in light of increasing global warming and frequent drought scenarios. This species presents a number of novel physiological mechanisms for drought resistance, as well as putatively related key genetic characteristics for the improvement of crops and pastures.

## Author Contributions

J-JX, R-JC, S-MW, and T-MH planned and designed the research. H-YC, W-PB, R-CY, and P-ZY performed the experiments. J-JX analyzed the data. J-JX and R-JC wrote the manuscript. All authors read and approved the final manuscript.

## Conflict of Interest Statement

R-JC was employed by company Noble Research Institute LLC. The remaining authors declare that the research was conducted in the absence of any commercial or financial relationships that could be construed as a potential conflict of interest.
