# Supplementary material for: Sodium-Related Adaptations to Drought: New Insights From the Xerophyte Plant Zygophyllum xanthoxylum
Source: Front Plant Sci. 2018 Nov 20;9:1678. doi: 10.3389/fpls.2018.01678 (PMC6255947; doi:10.3389/fpls.2018.01678)
Supplement: Supplementary file 1 [file Table_1.DOCX]

|  | **Number of stomata on adaxial epidermis** | | | | **Number of stomata on abaxial epidermis** | | | |
| --- | --- | --- | --- | --- | --- | --- | --- | --- |
| **Treatments** | Control | | NaCl | | Control | | NaCl | |
| **Stomata status** | Open | Partially closed | Open | Partially closed | Open | Partially closed | Open | Partially closed |
| Scanning Image 1 | 2 | 1 | 0 | 2 | 5 | 0 | 0 | 2 |
| Scanning Image 2 | 1 | 1 | 0 | 2 | 6 | 1 | 0 | 1 |
| Scanning Image 3 | 2 | 0 | 0 | 2 | 4 | 0 | 0 | 1 |
| Scanning Image 4 | 2 | 0 | 0 | 9 | 4 | 0 | 0 | 5 |
| Scanning Image 5 | 2 | 0 | 0 | 3 | 4 | 0 | 0 | 1 |
| Scanning Image 6 | 1 | 0 | 0 | 6 | 5 | 0 | 0 | 1 |
| Scanning Image 7 | 1 | 4 | 1 | 4 | 4 | 0 | 0 | 4 |
| Scanning Image 8 | 3 | 2 | 1 | 5 | 4 | 0 | 0 | 6 |
| Scanning Image 9 | 3 | 2 | 0 | 6 | 5 | 1 | 0 | 2 |
| Scanning Image 10 | 7 | 1 | 0 | 3 | 3 | 1 | 0 | 4 |
| Scanning Image 11 | 4 | 0 | 0 | 3 | 5 | 0 | 0 | 4 |
| Scanning Image 12 | 5 | 0 | 0 | 4 | 5 | 0 | 0 | 2 |
| Scanning Image 13 | 2 | 2 | 0 | 3 | 7 | 0 | 1 | 3 |
| Scanning Image 14 | 4 | 3 | 2 | 3 | 4 | 1 | 2 | 2 |
| Scanning Image 15 | 4 | 4 | 0 | 5 | 7 | 0 | 0 | 3 |
| Scanning Image 16 | 4 | 2 | 1 | 3 | 4 | 0 | 0 | 4 |
| Scanning Image 17 | 4 | 2 | 0 | 4 | 2 | 0 | 1 | 2 |
| Scanning Image 18 | 3 | 3 | 0 | 4 | 1 | 0 | 0 | 4 |
| Scanning Image 19 | 7 | 0 | 0 | 4 | 1 | 0 | 0 | 5 |
| Scanning Image 20 | 2 | 1 | 0 | 4 | 2 | 0 | 0 | 4 |
| Scanning Image 21 | 3 | 1 | 0 | 3 | 3 | 1 | 0 | 2 |
| **Total** | 66 | 29 | 5 | 82 | 85 | 5 | 4 | 62 |
| **Proportion** | 69.5% | 30.5% | 5.7% | 94.3% | 94.4% | 5.6% | 6.1% | 93.9% |

**Table S1** Proportion of open and partially closed stomata in leaf adaxial and abaxial epidermis based on SEM results. The samples for this experiment were third- and fourth-node leaves from 2-week-old *Z. xanthoxylum* seedlings treated with 50m mM NaCl or without (control) for seven days.
